# An investigation of the influence of skin colour on the perception of femininity, masculinity and likeable

**DOI:** 10.3389/fpsyg.2022.1044505

**Published:** 2022-12-08

**Authors:** Mengmeng Wang, Jingzhe Zhang, Jiapei Chen, Liyi Zhang

**Affiliations:** ^1^School of Design, Jiangnan University, Wuxi, China; ^2^School of Artificial Intelligence and Computer Science, Jiangnan University, Wuxi, China

**Keywords:** skin colour, facial image, femininity, masculinity, likable

## Abstract

Facial skin colour, a key factor related to impressions, is widely used by CG character designers to build characters with different storylines. The previous research provided essential suggestions for creating an attractive facial image. However, the suggestions of the prior research are insufficient for building the characters to resonate with the current public, especially young people. The present study investigates the influence of skin colour (whiteness and hue angle) on the femininity, masculinity and likableness perception of Chinese female and male images. A psychophysical experiment was carried out to investigate these relationships. The categorical judgement results reveal that whiteness significantly impacted the feminine-masculine perception of the Chinese male image and the likableness perception of the Chinese female and male image. This connection between the whiteness and likability of the male facial image could be related to the beauty trends in the last decade. The hue angle only significantly influenced the likability perception of the Chinese female image. This result is agreed with past research in the same area.

## Introduction

Skin is a vital facial feature of the human face. Its colour reflects the ethnicity, habitat and health of an individual subject (Alam et al., 2009). With a growing interest in computer-generated (CG) characters, like virtual idols, how to tune the skin colour to build a character with an expected perception has become a key issue for designers. Previous studies suggested that skin colour can be related to the attractiveness, health and youth of the facial image. The female with homogeneous skin tone appeared younger, healthier, and more attractive than the others (Fink et al., 2001; Fink and Matts, 2008). The female with reddish skin colour was perceived as healthier and more attractive (Fink et al., 2001). For the observers from different ethnic groups, the experimental results of African and Caucasian observers showed that both ethnicities with higher yellowness in skin colour were perceived as healthier (Stephen et al., 2009, 2011). A similar experiment was carried out with 30 Caucasian images. The observation results of 16 Caucasians showed that the one with healthy-look skin colour was also perceived as attractive (Whitehead et al., 2012). Recent research used the facial images in the Liverpool Leeds skin colour database (LLSC) to investigate the patterns between skin colour and the three impression attributes. Models to predict the strength of these perceptions were proposed (Lu et al., 2021). This research revealed the correlation between skin colour and perceived impressions, and demonstrated in a numeric way to evaluate the perceptions. The cultural background of the observers was also found to be a factor that influenced the preference for the faces (Lu et al., 2022). The Chinese observers were found to be more relied on skin colour to judge the preference of the facial images than the Caucasian observers.

Research on skin colour preference provided a numeric guide for the CG character’s skin colour tunning. The preferred skin colours were quantified in terms of CIELCh, which can be directly used to tunning the skin colours on the major character design software. These research use numbers to prove that Caucasian females prefer a more saturated and yellower skin colour than the original (Bartleson, 1960; Zeng and Luo, 2009). Oriental females prefer a “whiter” skin colour which is redder and has a higher lightness value than the originals’ (Ashikari, 2005). Another research further studied the “white skin colour” and found that the skin colour with a lower chroma was perceived as whiter than the one with high chroma (Yoshikawa et al., 2012).

With growing awareness and understanding of the uncorrelation between gender identity and the assigned gender, many traditional concepts are changed, such as beauty and attractiveness. Early research found that the attractiveness or preference of male and female images was related to masculinity or femininity, respectively (Philips, 2004; Scott et al., 2010). But recent research on the sociocultural impact of LGBTQ+ beauty influencers on cosmetic consumption reflected that the current beauty concept might not be benchmarked by gender (Armstrong, 2020). For example, the “neutral sex look” of Chinese female celebrities and the feminine appearance of Chinese male celebrities have become trending in China in the last decade. The concept of femininity, masculinity and likeable faces for the Chinese, especially young people, can be different from before (Wen, 2021). The growing number of male makeup influencers and the rising consumption of male makeup products in China reveal a shift in the stereotype of makeup and the general attitude to the concept of feminine and masculine beauty (Lau, 2018). This situation also indicated that the previous research on skin colour and attractiveness/preference faces might not provide sufficient support for the CG character designers to build a resonating facial image with the current Chinese young people. Therefore, new research on the relationship between skin colour and attractiveness is required.

Previous studies outline a critical role of skin colour to be investigated with the perception of facial images. The relationship between gender expression and beauty was changed as the uncorrelation between gender identity and the assigned gender was widely aware and started to be accepted. In this study, we investigated the femininity, masculinity and the likeableness of the facial image with skin colour as the independent factor. The results can help to reveal the attitude of young Chinese people toward gender expression and beauty concepts. Also, they can provide suggestions to the CG designer to tunning the skin colour of characters to achieve emotional resonance.

## Experimental design

### Stimuli

Two facial images, including a Chinese female (OF) and a Chinese male (OM), were selected from the Liverpool Leeds Skin Colour database (LLSC) to generate the stimuli that only differed in skin colour. LLSC contains the facial images and skin colour data of 188 subjects from four ethnicities, including Caucasian, Chinese south Asian and African (Xiao et al., 2017; Wang et al., 2018). The characterisation information and the measurement settings of the measurement instruments were also included. Two instruments were used to accumulate the skin colour data, a telespectroradiometer and a spectrophotometer. In this study, the measurement results from the spectrophotometer were used. These stimuli were generated with two stages of image processing. In the first stage, the skin area was segmented from the facial image. The texture of the skin area was extracted and stored. In the second stage, the texture was restored to the skin area with a selected skin colour which replaced the original colour. This new skin area was added to the facial image to generate a stimulus.

A threshold-based segmentation algorithm was used to segment the skin area (Jones and Rehg, 2002; Al-Tairi et al., 2014). The RGB values of the skin colours from the LLSCD were used to gain the segmentation threshold. Based on this threshold, a mask was generated to separate the skin area from the image. Here, the non-skin area included the facial features, such as the eye, eyebrows and lips. Note that, as the sclera of the eyes were hard to totally separate from the skin area, Adobe® Photoshop CS6 was used to mark these sections out manually in this study.

The skin texture extraction algorithm was adapted from the image processing method proposed by Young and Liu (1986). The CIELAB (D65, 10 degrees) colour space was used for image processing. Here, the skin texture is considered as the variation of the L^*^, a^*^and b^*^ at each pixel, which is against the mean L^*^, a^*^and b^*^ values of the whole skin area.

The skin colours investigated in this study were selected based on the distribution of the human skin in whiteness and CIE hue angle plane (D65, 10 degrees). The whiteness was calculated using the formula proposed in our previous research (Wang and Xiao, 2019), as shown in equation 1.


(1) $$L^{*}=\frac{L_{\text{Sub}}^{*}-\beta }{C_{\text{ab},\;}^{*}{\;}_{\text{sub}}}C_{\text{ab}}^{*}+\beta$$

where $L_{\text{Sub}}^{*}$ and $C_{\text{ab}}^{*}{\;}_{,\;\text{sub}}$ are the average lightness and chroma value of the skin colour from the LLSCD. β is the optimised coefficient. For OF and OM, the $L_{\text{Sub}}^{*}$, $C_{\text{ab},\;}^{*}{\;}_{\text{sub}}$ and β values were listed in Table 1.

**Table 1:** The $L_{\text{Sub}}^{*}$, $C_{\text{ab}}^{*}\text{sub}$ and β of OF and OM.

	$L_{\text{Sub}}^{*}$	$C_{\text{ab}}^{*}\text{sub}$	β
OM	63.74	21.38	76.0
OF	66.83	17.99	76.0

Twenty skin colours were selected to investigate in this study. The ranges of the selected skin whiteness were from 50 to 80 with 10 intervals. The chosen hue angles range from 30 to 70 with 10 intervals. In total, 40 images were examined in this experiment. Four images were randomly selected from these 40 images to be used as the repeat test to determine the variation of the observers’ judgement.

### Experimental procedure

**Figure 1:**
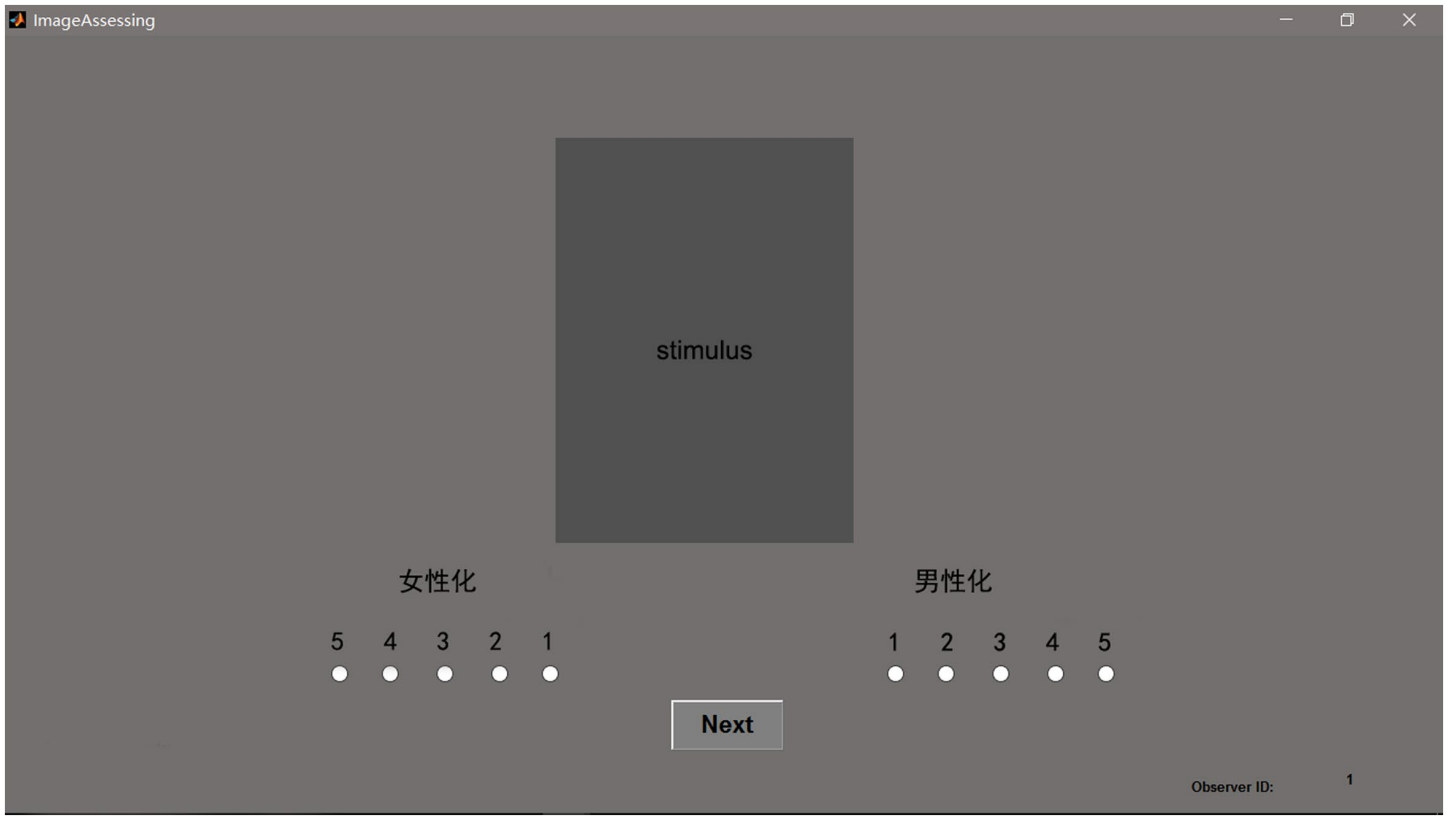
The experimental interface.

A force-choice categorical judgement method was used in this study. The stimulus was displayed at the centre of the screen with a 50% neutral colour background, as shown in Figure 1. The attribute was displayed below the stimulus in a random order for each stimulus. Two bipolar attributes, feminine-masculine and likeable-dislikeable, were used here. The participant was asked to select the term that was close to his/her perceived impression of the image first. Then scored it on a 5-point scale. In this experiment, score 5 represented a strong correlation between the term and the image, and score 1 represented a weak correlation. Forty Chinese subjects, including 20 Chinese females and 20 Chinese males, average age of 25.31, SD = 4.12, volunteered to participate in this experiment. Only the Chinese observers were invited to participate in this study, as cultural background could be a factor in influencing the judgement results. The colour vision of the participants was checked by using Ishihara’s Test for Colour Deficiency (24 plates) book under the simulated D65 illumination. All participants passed the test. Then they stayed in the dark environment for 5 min of adaptation. A training test aimed at assisting the observer in getting familiar with the experimental interface was conducted first. This training test included two sets of images and two attributes. The images used in the training test differed from those in the formal experiment. After the training test, a formal investigation was carried out.

### Statistical analysis

The intra-and inter-observer variation was evaluated through the Root Mean Square (RMS) of the repeat stimuli results and the 40 stimuli results, respectively. All the experimental results were converted into a z-score by adopting Case V of Thurstone’s Law of Comparative Judgements (Thurstone, 1927). The raw data of the experiment were first converted into a frequency matrix. Then the cumulative probability matrix can be achieved from the cumulative sum of the values in the frequency matrix. Based on the inverse standard normal cumulative distribution of the cumulative probability matrix, the z-score matrix can be gained. The relationship between the skin colour and the feminine-masculine and likeable-dislikeable were determined by using ANOVA. The correlation between the feminine-masculine and likeableness were examined by using the Pearson’s correlation coefficient. Both ANOVA results and Pearson’s correlation coefficient results were gained by using IBM SPSS Statistic 25 software.

## Results and analysis

### Intra–observer and inter–observer variation

The intra-observer and inter-observer variations were determined through Root Mean Square (RMS), as listed in Table 2. The intra-observer variation of the two attributes was similar and smaller than the inter-observer variation. The inter-observer variation of the feminine-masculine is larger than the likeable-dislikeable, which indicates that the variation between the observer is larger in judging the feminine-masculine than the likeable-dislikeable.

**Table 2:** The intra-and inter-observer variation (WD%).

	Feminine-masculine	Likeable-dislikeable
Intra-observer	1.59	1.55
Inter-observer	5.40	3.59

### The feminine–masculine and likeable-dislikeable facial skin colour

**Figure 2:**
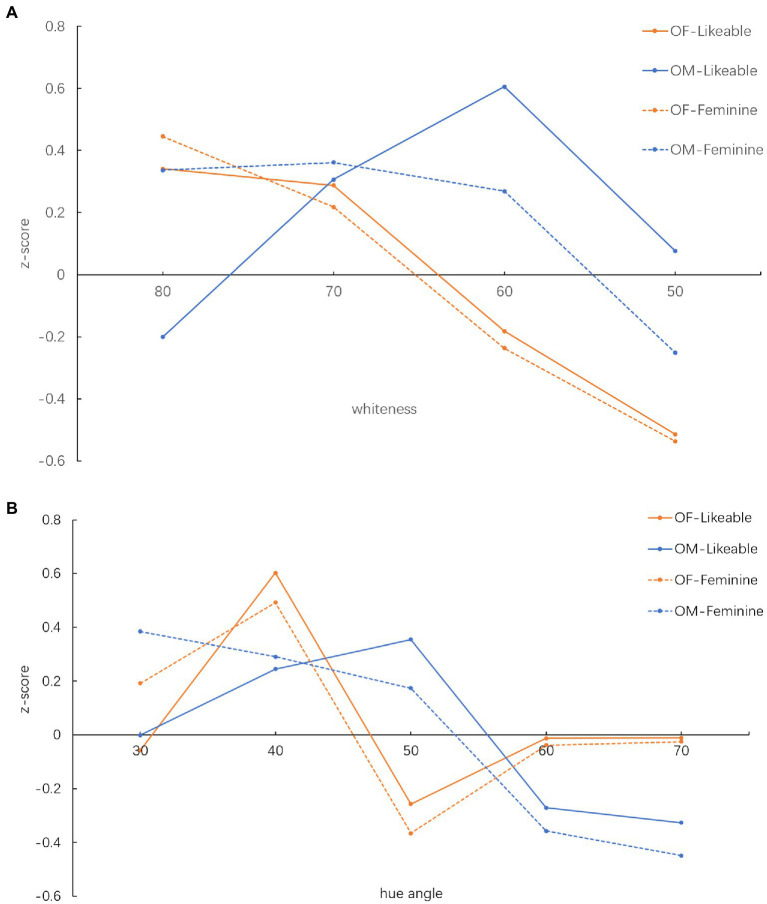
The z-score of OF and OM images with different **(A)**, whiteness and **(B)**, hue angle.

The score in the raw experimental data cannot represent an even interval score of the observer’s judgement. So, the raw experimental data were converted into z-scores for analysation. The variation of the z-scores at different skin whiteness and hue angle values for the two bipolar attributes can reflect the change of the perception at each stimulus. The judgement results of two bipolar attributes at the same stimulus can reflect the connection between the feminine-masculine and likeableness. The z-scores of each stimulus were calculated to determine the strength of the correlation between the skin colour and the attribute. The positive and the negative z-scores represent the perceived femininity/likeable and the perceived masculinity/dislikeable from the image, respectively. The z-scores of each whiteness and hue angle value were calculated and illustrated in Figure 2. The shape of the OF-feminine and OF-likeable trendlines are similar. A negative correlation direction can be observed from the trendline of OF-likable and OM-likeable, also the OM-likeable and OM-feminine, as shown in Figure 2A. OF-feminine and OM-feminine trendlines show a positive correlation when whiteness is the independent factor.

For both OF and OM images, the feminine of the facial image increased while whiteness increased. Both OM and OF with whiteness equal to 80 and 50 were perceived as the most feminine and the most masculine, respectively. The OF with hue angle equals to 40°perceived as the most feminine. The most feminine OM, with a hue angle of 30, is redder than the OF. The OM with a hue angle equal to 70°was perceived to be the most masculine. The OF is perceived as the most masculine when the hue angle equals 50. Note that this hue angle value was perceived as the most likeable for OM. The feminine-masculine and likeable-dislikeable of the OF showed a bipolar change at hue angles equal to 40°and 50. These showed that observers might be sensitive to the hue angle changes of the OF image within this range. The OM was perceived as the most likeable when the skin whiteness equals 60. The most likeable OF is whiter than the OM, which whiteness value equals 80. The OM did not perceive as more likeable when the whiteness decreased 10.

### ANOVA

The significance of the bipolar attributes judgement results at different whiteness or hue angle levels was examined through ANOVA. The analysis results can determine the influence of skin whiteness or hue angle on the feminine, masculine or likeableness perception, which can be used to infer the driving of the judgements of feminine-masculine and likeableness. Welch’s ANOVA and Games-Howell *post hoc* pairwise comparison was used to analyse the experimental results. The analysations were conducted on the dependent variables femininity-masculinity and likeable-dislikeable. The hue angle and the whiteness were the factors of the stimulus. Here, the interaction effect of the two independent factors is not the interest of the current study. The one-way ANOVA of each factor was carried out. All significant values were reported at *p* ≤ 0.05.

#### Feminine–masculine

**Figure 3:**
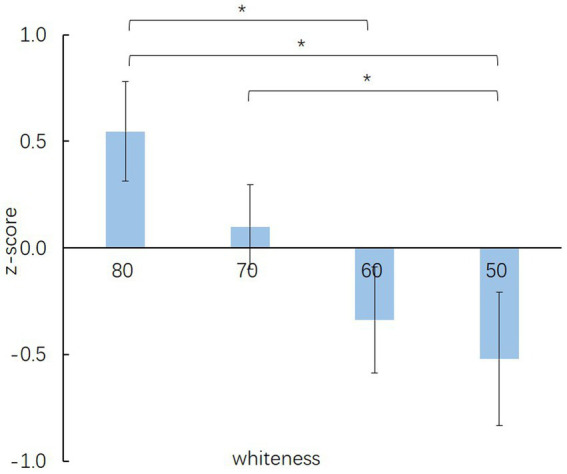
Mean rating (±SE) of the OM stimuli with different whiteness levels at feminine-masculine. Asterisks indicate statistical significance at *p < 0.05 (Games-Howell corrected).

The Welch’s ANOVA result shows that whiteness has a significant influence on the feminine-masculine perception of OM $F_{\text{Welch}}\left(3,10.695\right)=5.753,$ p<0.05, $\eta^{2}=0.468.$ But it does not significantly impact the femininity-masculinity of OF, $F_{\text{Welch}}\left(3,10.707\right)=1.765$, p>0.05, $\eta^{2}=0.213$. The hue angle has no significant impact on the feminine-masculine perception of OM, $F_{\text{Welch}}\left(6,7.968\right)=0.296$, p>0.05, $\eta^{2}=0.058,$ and OF, $F_{\text{Welch}}\left(5,9.126\right)=0.967$, p>0.05, $\eta^{2}=0.151.$ Figure 3 illustrates the Games-Howell *post hoc* test results of the OM with different whiteness. A descending in z-scores while the whiteness value decreases can be found in this figure. Games-Howell *post hoc* test results indicated that the OM with whiteness equal to 80, (0.5469±0.23349), is significantly more feminine than the OM with whiteness equal to 60, (−0.3386±0.24911), and 50, (−0.5212±0.31274). The OM with whiteness equal to 70, (−0.0999±0.19782), is significantly more feminine than the OM with whiteness equal to 50. The z-scores of the OM images with whiteness intervals equal 10 showed insignificant differences. A significant difference was found between the OM images with whiteness intervals equal to 20 or above.

#### Likeable–dislikeable

**Figure 4:**
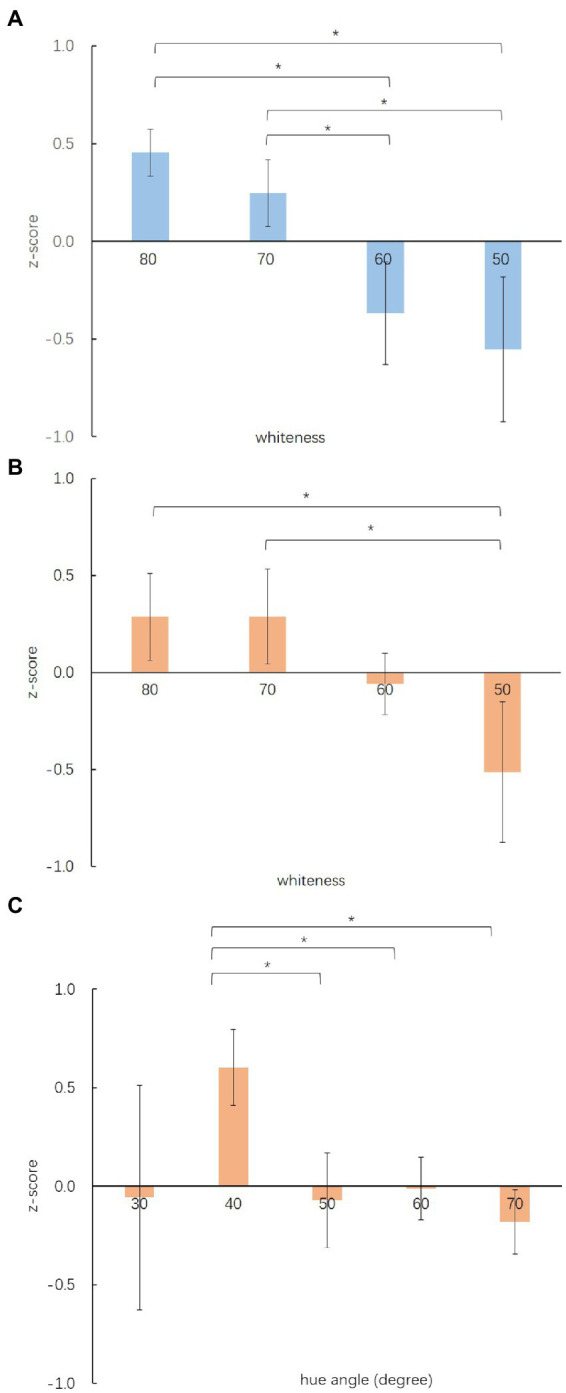
Mean rating (±SE) of **(A)** the OM images with different whiteness levels, **(B)** the OF images with different whiteness levels, and **(C)** the OF images with different hue angles at likeable and dislikeable. Asterisks indicate statistical significance at *p < 0.05 (Games-Howell corrected).

The Welch’s ANOVA result shows that whiteness has a significant effect on the likeable perception of OM, $F_{\text{Welch}}\left(3,10.010\right)=3.886$, p<0.05, $\eta^{2}=0.389$, and OF, $F_{\text{Welch}}\left(3,10.962\right)=1.468$, p<0.05, $\eta^{2}=0.238$. The hue angle also has a significant impact on the perception of likeable for OF, $F_{\text{Welch}}\left(6,7.704\right)=1.043$, p<0.05, $\eta^{2}=0.125$, but not for the OM, $F_{\text{Welch}}\left(5,9.291\right)=2.041$, p>0.05, $\eta^{2}=0.171$. Games-Howell *post hoc* test results show that the OM with whiteness equal to 80, (0.4536±0.11992) and 70, (0.2455±0.17159), is significantly perceived as more likeable than the OM with whiteness equal to 60, (−0.3689±0.26132), and 50, (−0.5533±0.36953). The OF with whiteness equals 80, (0.2850±0.22320), and 70, (0.2874±0.24487), are significantly perceived as more likeable than the OF with whiteness equals 50, (−0.5144±0.36334). The OF with a hue angle equal to 40°, (0.6020±0.28684), is significantly perceived as more likeable than the OF with a hue angle equal to 50°, (−0.0709±0.33849), 60°, (−0.0124±0.15858), and 70°, (−0.1821±0.12556). Figure 4 illustrates the Games-Howell *post hoc* test results above. The blue and orange bars represent the results of OM and OF, respectively. For both OM and OF, the z-scores of the likeable-dislikeable descend while the whiteness decreases. For OF, the one with a hue angle more than 40°was significantly less in likableness.

**Figure 5:**
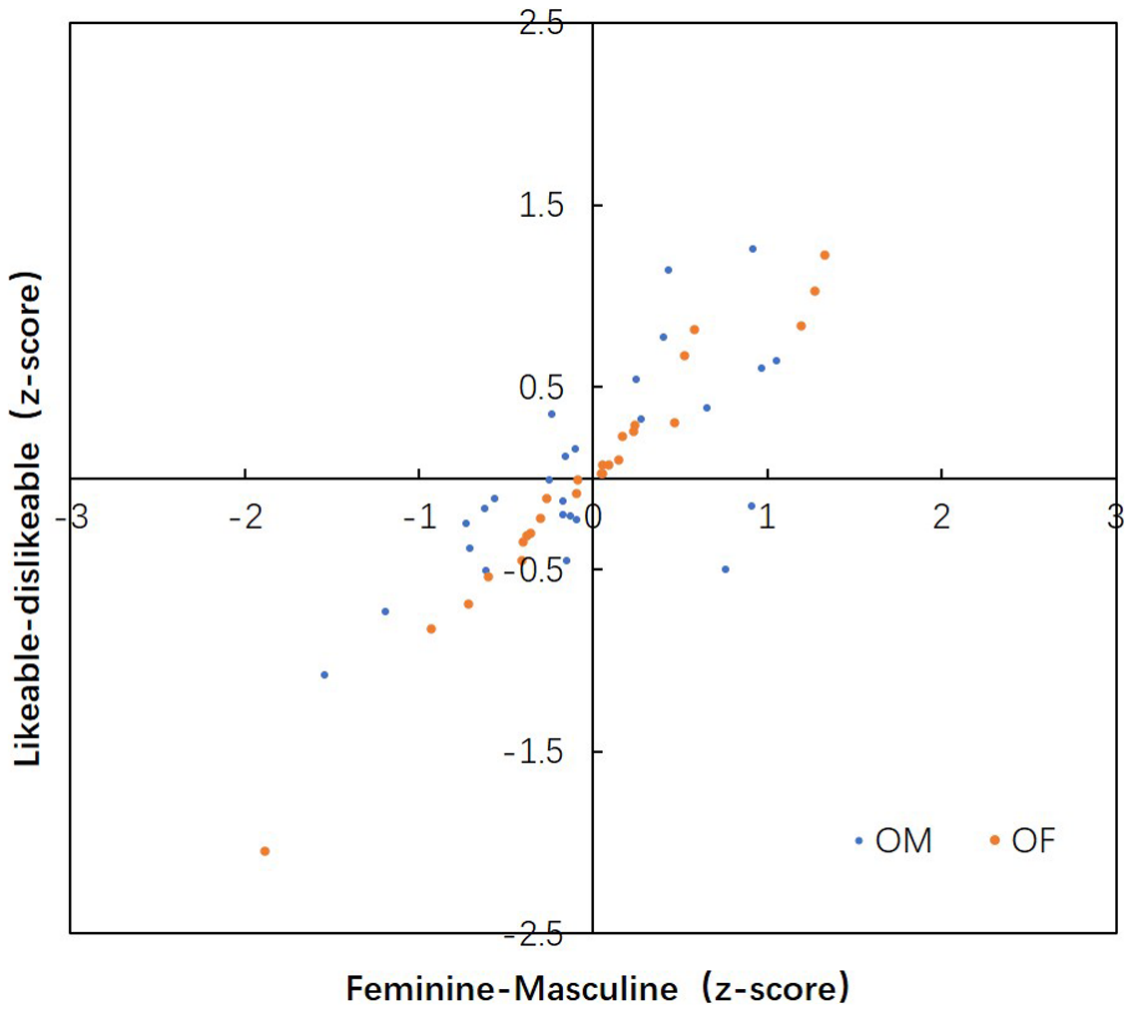
The correlation between the feminine-masculine and likeable-dislikeable.

#### Correlation between feminine–masculine and likeable–dislikeable

The association between the feminine-masculine and likeable-dislikeable was determined through Pearson’s correlation coefficient. Figure 5 illustrates the z-score distribution of the results from the female stimuli and male stimuli. A strong correlation between these two attributes infers a strong linear association, which indicates the value of one attribute can be predicted by another. For this research, a strong correlation indicated the judgement results of likeable-dislikeable can be predicted from feminine-masculine, which infers that the results of feminine-masculine and likeable-dislikeable are connected closely. It can be a supportive statistic for further study in gender perception and likableness in facial image. The correlation coefficient of OF images and OM images were calculated, respectively. For OF, the correlation coefficient *r* = 0.95, which indicates the likeable-dislikeable have a strong positive linear correlation to the feminine-masculine (*r* > 0.6). For OM images, the correlation coefficient *r* = 0.503, which shows that the likeable-dislikeable have a positive weak linear correlation to the feminine-masculine.

## General discussion

This study investigated the femininity, masculinity and likeableness of the facial image when skin colour is the independent factor. Twenty skin colours and images of both genders, which were selected from the LLSC database, were used in this study. The results showed that the perceived femineity, masculinity and likableness were changed with the whiteness and hue angle. The whiteness was found to be significantly influenced the feminine-masculine perception of OM, and the likeable perception of the OF and OM. The hue angle was found to only influence the likeable perception of the OF significantly. For the linear correlation, the feminine-masculine and likeability-dislikeable have a strong positive correlation for judging the OF image. For the OM, the correlation between two bipolar attributes was positive but weak.

Our ANOVA analysis shows that the feminine-masculine perception of the OM is significantly influenced by skin whiteness. The OM with whiter skin colour provided a feminine perception than the darker one. The androgyny or effeminate male beauty trend raised in the past decades. One of the methods to “soft” the masculine appearance at the time was to lighten the skin tone (Wen, 2021). In our experimental results, the feminine and likeable perception of the male image related to skin whiteness could be influenced by this “soften masculine method.” This trend of the “flower boy,” or “xiaoxianrou (小鲜肉)” in Chinese, triggered debates about the “proper” appearance or behaviours of a young male in 2018, which led to a fade of the effeminate male beauty trend. From the experimental results of likeable, we can find a clue of that trend fading, where the most likeable OM is darker than the most feminine perception OM. The correlation coefficient between the likeable and feminine-masculine of OM is positively correlated but has a weak correlation. This indicated that the likeable of the OM is ascending along with the increasing of the perceived feminine. However, the whiteness of this most likeable OM image is also perceived as feminine. We can infer from this result that the feminine perception of OM is still preferred by young Chinese people. Also, the femininity perception might be a part of the likeable judgement. But our study results are not sufficient to validate this hypothesis. We may carry out another experiment to determine the influence of the feminine perception on the likeable judgement for OM.

This study showed that skin colour significantly influenced the likability of OF images, which agreed with the previous research (Yoshikawa et al., 2012). The maximum z-score of these two attributes appeared at a hue angle equal to 40, and the minimum appeared at a hue angle equal to 50°, as shown in Figure 2B. This bipolar shift infers that the Chinese can be sensitive to the hue angle change in the skin colour at these two hue angles. The hue angles of the Chinese skin colour are majorly distributed with the ranges of 40–50 (Xiao et al., 2017). So the Chinese observers were more familiar with the judgement that was carried out between these two hue angles than the one out of the range. This might lead to the bipolar shift between two adjacent hue angles. These results also show that the Chinese observers preferred a reddish skin tone over the yellowish one, which agrees with the previous research in the east Asian preferred skin colour study (Yamamoto et al., 2003). In recent research, skin colour, especially the a^*^, L^*^ and the contrast of the a^*^ around brows and mouth area, were found to be the key predictor factor for facial preference (Lu et al., 2022). Our study agreed with this finding, which indicated that whiteness and redness are two important indices of facial preference.

The result of the preferred OF skin colour hue angle in this study also agreed with some previous research, where the hue angle equal to about 40 was found to be the most preferred (Yano and Hashimoto, 1997; Fan et al., 2011). But our results are less agreeable to Zeng and Luo (2009). The preferred skin colour of Zeng and Luo (2013) is yellower than the present study. But they claimed that the preferred skin colour is redder than the actual skin colour. The disagreement can be because their analysation is carried out on a^*^b^*^ plane. The variation caused by different L^*^ was not included. Note that, to make a valid comparison, only the OF results of this study are discussed. The agreement between the previous research and this study might indicate that the preference of the OF skin colour hue angle has been unvaried in the last decades. As limited research investigated male skin colour, the change of the preference in OM is difficulted to be determined.

## Data availability statement

The raw data supporting the conclusions of this article will be made available by the authors, without undue reservation.

## Ethics statement

The studies involving human participants were reviewed and approved by The ethic committee of Jiangnan University. The patients/participants provided their written informed consent to participate in this study.

## Author contributions

MW designed the study. JC and LZ collected the data. MW and JZ analysed the data. All authors contributed to the article and approved the submitted version.

## Funding

This research was supported by Jiangsu Provincial Basic Research Programme Natural Science Foundation to MW (BK20200613).

## Conflict of interest

The authors declare that the research was conducted in the absence of any commercial or financial relationships that could be construed as a potential conflict of interest.

## Publisher’s note

All claims expressed in this article are solely those of the authors and do not necessarily represent those of their affiliated organizations, or those of the publisher, the editors and the reviewers. Any product that may be evaluated in this article, or claim that may be made by its manufacturer, is not guaranteed or endorsed by the publisher.
